# Mobile Brain/Body Imaging (MoBI) of Physical Interaction with Dynamically Moving Objects

**DOI:** 10.3389/fnhum.2016.00306

**Published:** 2016-06-27

**Authors:** Evelyn Jungnickel, Klaus Gramann

**Affiliations:** ^1^Department of Psychology and Ergonomics, Biological Psychology and Neuroergonomics, Institute of Psychology and Ergonomics, Berlin Institute of TechnologyBerlin, Germany; ^2^Center for Advanced Neurological Engineering, University of CaliforniaSan Diego, CA, USA

**Keywords:** mobile brain/body imaging, EEG, embodied cognition, independent component analysis, P300, oddball paradigm, MoBI

## Abstract

The non-invasive recording and analysis of human brain activity during active movements in natural working conditions is a central challenge in Neuroergonomics research. Existing brain imaging approaches do not allow for an investigation of brain dynamics during active behavior because their sensors cannot follow the movement of the signal source. However, movements that require the operator to react fast and to adapt to a dynamically changing environment occur frequently in working environments like assembly-line work, construction trade, health care, but also outside the working environment like in team sports. Overcoming the restrictions of existing imaging methods would allow for deeper insights into neurocognitive processes at workplaces that require physical interactions and thus could help to adapt work settings to the user. To investigate the brain dynamics accompanying rapid volatile movements we used a visual oddball paradigm where participants had to react to color changes either with a simple button press or by physically pointing towards a moving target. Using a mobile brain/body imaging approach (MoBI) including independent component analysis (ICA) with subsequent backprojection of cluster activity allowed for systematically describing the contribution of brain and non-brain sources to the sensor signal. The results demonstrate that visual event-related potentials (ERPs) can be analyzed for simple button presses and physical pointing responses and that it is possible to quantify the contribution of brain processes, muscle activity and eye movements to the signal recorded at the sensor level even for fast volatile arm movements with strong jerks. Using MoBI in naturalistic working environments can thus help to analyze brain dynamics in natural working conditions and help improving unhealthy or inefficient work settings.

## Introduction

Studying human brain dynamics accompanying natural cognition (Gramann et al., 2014) works best by studying the brain under naturalistic conditions. The embodied cognition paradigm claims that the body’s interactions with the world are an essential root of cognitive processes (Wilson, 2002). Thus it appears that perception and action should both be considered when studying cognitive processes and their neural basis. However, conventional neuroimaging studies consider electrical potentials generated by eye movement or muscle activity during physical movements as artifacts that have to be avoided not to contaminate the signal of interest. This view led to experimental setups that restrict participants’ mobility and require them to sit still or lie even in tasks that would require standing or moving (Makeig et al., 2009; Gramann et al., 2011, 2014). These constraints are changing the way information is perceived and processed by the human agent as becomes obvious, for example, with respect to the integration of proprioceptive and vestibular information (Gramann, 2013). This kind of idiothetic information is absent when movement is restricted or altered in case the body orientation differs from its natural state for a particular task. Following the embodied cognition approach those alterations will change the concurring cognitive processes and thereby lead to different brain activity.

Neuroergonomics as the scientific study of the human brain in relation to performance at work and everyday settings (Parasuraman, 2003) is faced with the challenge to investigate the brain dynamics in environments that require physical interaction of the operator with a system. New insights into brain activity during physical human-machine interaction allow for the improvement of systems to adapt to the operators’ physical and cognitive resources (see e.g., Wascher et al., 2016; Mijovic et al., 2016). However, traditional brain imaging approaches do not allow for any kind of movement (Makeig et al., 2009; Gramann et al., 2011). Mobile brain/body imaging (MoBI), in contrast, is a general research approach that embraces a variety of (the best fitting) hardware and software solutions to record and analyze brain dynamics in actively behaving participants. Lightweight and mobile sensors like electroencephalography (EEG) or near infrared spectroscopy (fNIRS) agree with experimental paradigms using a MoBI approach to study the brain and body dynamics that accompany natural cognition and behaviors including physical interaction with an environment (Mehta and Parasuraman, 2013; Gramann et al., 2014). While fNIRS provides relatively high spatial resolution of a restricted cortical surface, this methods lacks the high temporal resolution that is desirable when investigating fast cognitive processes. EEG provides the necessary temporal resolution but has only limited spatial resolution. However, recent investigations using MoBI have demonstrated that equivalent dipole reconstruction of independent components (ICs) as decomposed by independent component analysis (ICA) allow for reconstructing the origin of EEG activity with reasonable spatial accuracy (Gramann et al., 2010a; Acar and Makeig, 2013). In conclusion, mobile EEG allows for an investigation of cognitive processes in working environments with high temporal resolution and with sufficient spatial resolution to allow for conclusions regarding the underlying cortical sources and their neuroanatomical function. Such a MoBI approach no longer considers eye movements and muscle activity as artifacts but as aspects of cognitive activity associated with the accomplishment of a task (Gramann et al., 2010a). By using high density EEG recordings synchronized with motion tracking of participant’s movements and data-driven analyses methods it overcomes existing imaging restrictions and enables participants to behave more naturally (Makeig et al., 2009; Gramann et al., 2011, 2014).

First MoBI studies investigated participants walking and running on a treadmill and clearly demonstrated that brain activity can be analyzed under such conditions (Gramann et al., 2010a; Gwin et al., 2010, 2011). However, walking is a highly symmetric recurrent behavior that does not include fast movements associated with jerk. Stereotyped movements like walking further allow for extracting templates of artifacts based on recurrent movement patterns (Gwin et al., 2010). It is important to investigate to what extent MoBI can be used to measure and analyze brain dynamics during nonstereotyped and aperiodic behaviors that include sudden orientation movements or manual interaction with dynamic systems. First, such an approach could be used to determine how much traditional brain imaging results restricting participants’ movements deviate from results in actively moving, more naturally behaving participants. Secondly, if feasible, such an approach would significantly increase the number of conceivable neurocognitive studies especially in the fields of physical ergonomics and in human-machine interaction that require physical manipulation. Insights gained from MoBI studies comprising natural recurrent and non-stereotyped movements would thus open up new vistas for investigating cognition and action within the field of Neuroergonomics and beyond.

This study investigated the feasibility of MoBI during physical interaction with a dynamic system based on non-stereotypical fast movements. The setup mimicked real-world working environments that require physical interaction in a dynamically changing system. Dynamic changes in the system were simulated using a three-stimulus visual oddball paradigm (Grillon et al., 1990) with participants reacting either by simple button presses or by pointing at the moving stimulus. We examined whether it is possible to record and analyze an event-related P3 component during rapid pointing movements that include strong eye movement and neck muscle activities. To this end we compared event-related potentials (ERPs) at the sensor level with ERPs back projected from ICs that decomposed the sensor data into maximally statistically independent time source series using ICA. By separating brain processes from activity generated by muscles and eye movement and comparing these to the scalp recorded potential allowed for a direct comparison and evaluation of the feasibility of standard sensor based analyses approaches during active pointing movements. In addition, isolation of brain related activity patterns and their contribution to the surface signal allowed for a quantification of how much certain ICs representing brain processes contributed to the surface signal.

## Materials and Methods

### Participants

Data was collected from 15 healthy right-handed adult volunteers (7 females, 8 males) with a mean age of 26.1 years (*σ* = 2.9). All participants had normal or corrected to normal vision, none reported a history of neurological disease and all provided written informed consent before the experiment in compliance with the standards as defined in the Declaration of Helsinki. The study was approved by the local ethics committee of the Institute of Psychology and Ergonomics of the Berlin Institute of Technology according to the guidelines of the German Psychological Society. Volunteers were compensated 12 €/h for their participation. Due to technical issues the behavioral data of three participants had to be excluded from further analysis and all results reported are based on the final group of 12 participants.

### Experimental Design and Procedure

**Figure 1:**
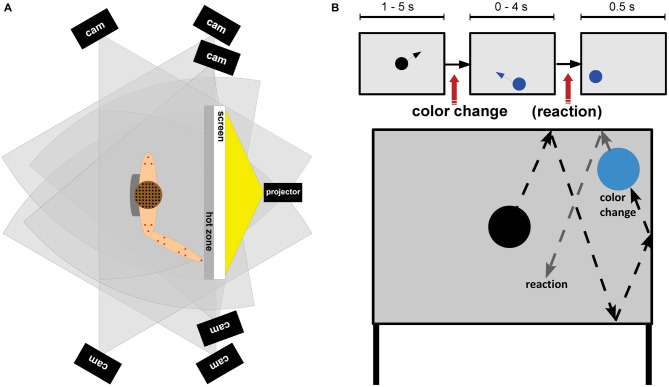
**(A)** Experimental setup: top view of a participant standing in front of the screen. The setup comprised a motion capture system with six cameras (black rectangles) and 16 emitters (red dots), an EEG system with 156 wireless actively amplified electrodes (black dots), and the transmission system placed in a backpack (gray). **(B)** Task design. Top row: a black sphere moved over the projection screen and bounced off the walls of the screen. After changes to the target color participants responded according to the response condition either with a button press (button press condition) or a pointing movement towards the sphere (physical pointing condition). After a response or 4 s after a color change the sphere stopped and remained on the screen for 500 ms. Subsequently the next trial started. Bottom row: example of a trajectory of the sphere changing to the target color.

Participants stood in front of a projection screen (W × H: 1.2 m × 1.0 m) with a light gray background placed one arm length in front of them (Figure 1). Participants had to attend to a three-stimulus visual oddball paradigm and were asked to react to color changes of a moving sphere by either pointing to the stimulus with their right index finger (physical pointing condition) or pressing a response button (button press condition) on a Bluetooth remote (Logitech wireless presenter R400, Logitech, Apples, Switzerland). The response conditions were blocked and block order was counterbalanced across participants. Each response condition consisted of five blocks with 50 trials each. Breaks between blocks within each response condition were adapted to the participants needs.

Every trial began with a black sphere (ø14 cm) moving from the middle of the screen in a randomly chosen direction and being reflected from the borders of the projection screen. Color changes took place uniformly randomized between 1 and 5 s after onset of a trial. A change from black to blue indicated a target stimulus (15%), a change to green indicated a distractor stimulus (15%), and a change to yellow indicated a standard stimulus (70%). Participants were instructed to react as fast and correct as possible to the onset of the target color. After a response, or after 4 s in case no response was given, the sphere stopped moving and remained on the screen for 500 ms. Thus, the trial duration for correct non-target trials ranged from 5.5 to 9.5 s with an average duration of 7.5 s. For target trials the mean trial duration was shorter because button presses or pointing movements were executed before the 4 s time window closed. Thus, the duration of target trials depended on the response onset, movement speed and movement path. Altogether the experiment lasted about 1 h.

### EEG Recording

The EEG was recorded from 156 active electrodes referenced to Cz with a sampling rate of 500 Hz and band-passed from 0.016 Hz to 250 Hz (BrainAmps and Move System, Brain Products, Gilching, Germany). To allow for recording of neck muscle activity resulting from participants’ head movements, 28 electrodes were placed around the neck using a custom neck band (EASYCAP, Herrsching, Germany). The remaining 128 electrodes were placed on the head using an elastic cap with a custom design (EASYCAP, Herrsching, Germany). Electrode impedances were brought below 7 kΩ. Due to a technical problem the neck EEG data of one participant was not recorded. Individual electrode locations were recorded using an optical tracking system (Polaris Vicra, NDI, Waterloo, ON, Canada).

### Motion Capture Recordings

Motion was captured using six cameras tracking the position of 16 red active LEDs (Impulse X2 System, PhaseSpace Inc., San Leandro, CA, USA) placed on the shoulders, the chest, and the right arm as well as the right index finger of the participants. The motion tracking system generated a data stream containing *x*, *y*, and *z* location and a reliability value for each LED with a sampling rate of 480 Hz. Before each data acquisition the screen position and orientation was calibrated to align with the motion capture coordinate system.

All data streams, namely EEG, motion capture, events from the experimental protocol, and behavioral data, were synchronized and recorded using the Lab Streaming Layer Software (Kothe, 2014).

### Behavioral Analysis

In the physical pointing condition, online tracking of the LED on the participants’ right index finger allowed to stop sphere movement as soon as the distance between the LED and the projection screen was smaller than 10 cm (labeled “hot zone” in Figure 1). This information was also used to create corresponding event markers. The LED was placed 5 cm apart from the fingertip approximately over the proximal phalanx of the index finger. The distance of 10 cm was chosen to avoid damage to the setup due to impact of the participants’ finger with the screen. Because of occlusions the position of the finger LED was not recorded correctly in some trials and event markers were generated that did not match the movement profile of the participant. For the statistical analyses only trials with consistent event markers and motion tracking data of the right index finger were considered. This led to the exclusion of about 34.4% of the trials per participant in the physical pointing condition (range: 5.2–69.9%, *σ* = 21.7%) with the highest percentage of removals in standard ($\bar{x}$ = 38.0%) and distractor trials ($\bar{x}$ = 38.3%) that required no response. In these cases event markers indicated a movement even though in most cases the velocity profile did not indicate a response. In case of target trials on average only 13.1% were rejected.

To calculate velocity profiles from the motion capture data the MATLAB toolbox MoBILAB (Ojeda, 2011) was used. Occluded samples for each LED were interpolated by using spline interpolation and the data stream was smoothed by applying a 6 Hz low-pass zero phase distortion FIR filter before computing the velocity data. Subsequently the velocity profiles in the *z*-dimension of the LED placed on the index finger were analyzed with custom MATLAB scripts detecting pointing movements in the physical pointing condition on the basis of velocity peaks. To identify response movements, only the *z-axis* of the motion capture data was used indicating motions towards or away from the screen. This excluded smaller movements not related to the response. Based on velocity peaks defined as maximum positive deflections preceding and being followed by lower values, the onset, and offset of the corresponding movement were defined. For each color change the time window from 200 to 1800 ms after stimulus onset was selected to exclude movements unrelated to the stimulus response. As estimated from visual inspection only peaks with a velocity of at least 22% of the participants’ maximum finger velocity in the physical pointing condition were regarded. This excluded smaller jerks and other movements not related to the pointing behavior. The definition of the movement onset is important in this context since its time-lag to the color change was taken as response time and used for further statistical analysis. The earliest movement onset was defined as the time point with a velocity of 5% of the subsequent peak velocity. To allow for a more conservative comparison of response times in the physical pointing condition with response times in the button press condition, increasing percentage values (>5%) of the subsequent maximum peak velocity were analyzed. The resulting movement onset distributions were then compared to response time distributions in the button press condition where no velocity profiles or force time-series could be derived. Response time statistics were calculated by means of a one-way analysis of variance (ANOVA) with subsequent correction for multiple comparisons using honestly significant difference (HSD) contrasts (Tukey, 1949).

### EEG Analysis

#### EEG Data Preprocessing

**Figure 2:**
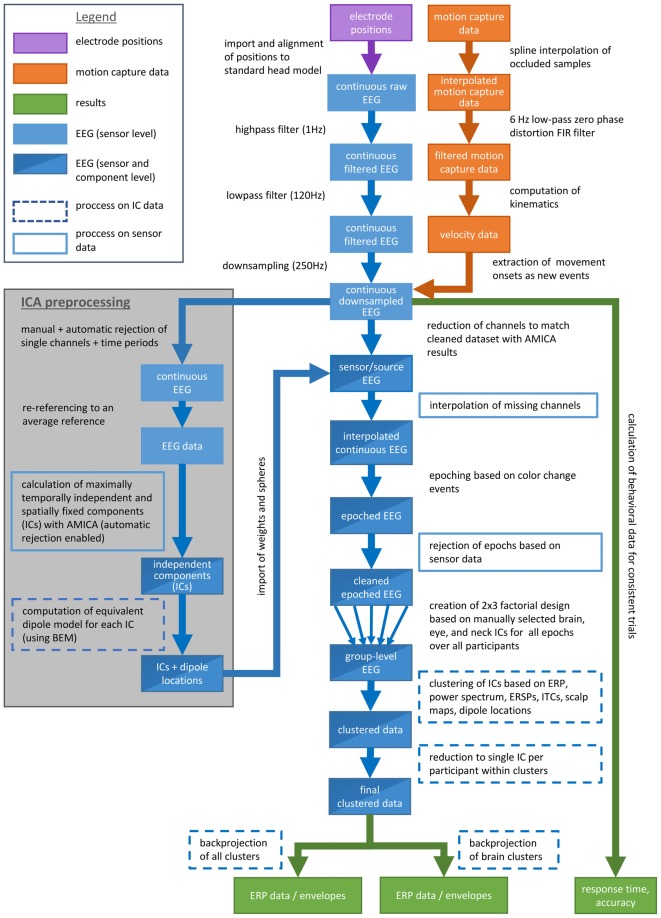
**Flow chart explaining the data processing pipeline**.

Data analysis was done by custom Matlab scripts based on the open source EEGLAB toolbox^1^ (Delorme and Makeig, 2004) . Figure 2 shows a flow chart explaining the whole data processing pipeline. The data was filtered using a high-pass filter (1 Hz) and a low-pass filter (120 Hz) and subsequently down sampled to 250 Hz. Single channels and time periods containing artifacts were removed by visual inspection of the data. Eye movements were not considered as artifacts. Artifact rejection was performed with an EEGLAB function automatically removing channels in case they contained zero activity for more than 5 s or revealed a correlation value below 0.6 with neighboring channels and time windows containing more than 30% noisy channels. On average, 132 EEG channels remained for further analyses (range: 114–142; *σ* = 8.1).

In a next step the data was re-referenced to an average reference and then parsed into maximally temporally independent and spatially fixed components (ICs; Makeig et al., 1996) using an adaptive ICA mixture model algorithm (AMICA; Palmer et al., 2006, 2008) which is a generalization of former ICA approaches as the infomax (Bell and Sejnowski, 1995; Lee et al., 1999a) and the multiple mixture approach (Lee et al., 1999b; Lewicki and Sejnowski, 2000). After the first iteration the model was trained for 10 iterations rejecting time windows with a likelihood below 4 standard deviations (SDs). For the remaining parameters the default settings were used (Palmer, 2016).

For each IC an equivalent dipole model was computed using a boundary element head model (BEM) based on the MNI brain (Montreal Neurological Institute, MNI, Montreal, QC, Canada) as implemented by DIPFIT routines (Oostenveld and Oostendorp, 2002). To this end corresponding landmarks (nasion, ion, vertex and ears) were aligned by rotating and rescaling each individually measured electrode montage. The use of an average head model decreases the accuracy of source localization and thus we refer to the approximation of the spatial origin of surface activity using the description “in or near” a specific structure. ICs primarily accounting for brain, eye or neck muscle activity were selected for further analysis based on their time courses, spectra, and scalp topographies as well as the location and residual variance of their corresponding dipoles. Dipoles placed outside of the head model were not further considered. This resulted in 594 remaining ICs for all participants with an average of 49.5 ICs per subject (range: 31–92, *σ* = 17.3, *∑* = 594). The weights and spheres returned from the AMICA decomposition were copied to the down sampled, high- and low-pass filtered continuous EEG data excluding the same channels that were excluded for ICA decomposition. Missing channels were interpolated.

#### EEG Group Level Analyses

The continuous data was epoched into 3 s long epochs with onset of a color change including a 1 s pre-stimulus baseline. Only epochs with correct responses were included in the study. Artifactual epochs containing fluctuations above 1000 μV or data values outside of 5 SDs on the sensor level were rejected in an iterative fashion keeping at least 95% of the total trial numbers per iteration. The remaining epochs ( $\bar{x}$ = 370.5 per participant, *σ* = 53.1) were subsequently combined into a study. The study comprised a 2 (response condition) × 3 (stimulus type) factorial design providing main effects for the two independent variables as well as their interaction.

Distances between all ICs were calculated with the weighted measures of ERP, power spectrum (for a frequency range of 3–75 Hz), event-related spectral perturbations (ERSPs), inter-trial coherences (ITCs), the components’ scalp maps and their equivalent dipole model locations using the EEGLAB preclustering function. For all measures (except dipole location with only three dimensions) a principal component analysis (PCA) reduced the dimensionality to the first 10 principle components. The resulting measures were normalized, weighted and combined into cluster position vectors. Dipole locations were weighted by a factor of 25 to promote spatially tight clusters and to compensate for its low dimensionality. ERSPs were weighted with a factor of 10 as they were assumed to express the most relevant time-varying information regarding the task. All other measures were weighted with the standard weighting of 1. Subsequently a PCA restricted the resulting cluster position vectors to a 10-dimensional subspace.

Clustering was done via a K-means algorithm implemented in EEGLAB with the number of clusters set to 36. By default, ICs with a distance of more than 3 SDs to the mean of any cluster centroid in joint measure space were assigned to an outlier cluster. The same was done manually for ICs if a cluster contained more than one IC of a participant relying on the same measures as for the calculation of the cluster position vectors. The residual variance of the equivalent dipole models of the remaining ICs was about 10.5% for all ICs representing brain processes (range: 1.3–47.7%, *σ* = 7.4%) and about 23.7% for all other ICs (range: 2.8–69.1%, *σ* = 14.3%). Overall, 302 ICs were assigned to the outlier cluster and 292 ICs were assigned to the other clusters (range: 20–30, $\bar{x}$ = 24.3, *σ* = 2.6 ICs per participant). Of those 292 ICs, 106 ICs revealed equivalent dipole locations within the gray matter of the head model (range: 7–11, $\bar{x}$ = 8.8, *σ* = 1.4 ICs per participant).

## Results

### Behavioral Data

**Figure 3:**
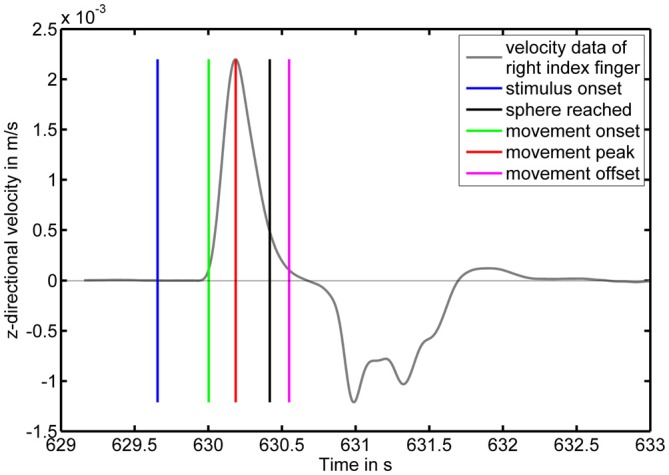
**Pointing movement velocity profile as a function of time with corresponding markers.** The *y*-axis displays the *z*-component of the velocity in m/s with positive values corresponding to motion towards the screen. The blue vertical line indicates a color change of the moving sphere to the target color. The green and magenta vertical lines indicate the movement onset and offset, respectively. The red vertical line indicates the velocity peak. The black vertical line indicates a distance between LED and projection screen below 10 cm.

An exemplary velocity profile for one physical pointing response with corresponding events derived from the velocity profile and the system generated markers is displayed in Figure 3 illustrating a typical pointing movement. In most cases, movements towards the screen were faster than the subsequent backward movements to the initial position.

**Figure 4:**
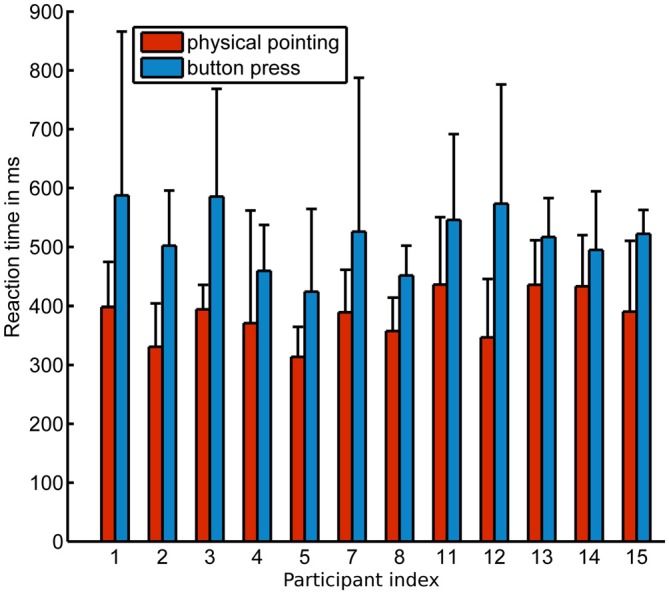
**Mean onset response times (threshold criterion 5% of subsequent max. velocity) for all participants in the physical pointing and button press condition.** *x*-axis displays the participant index, *y*-axis the response time in ms. Error bars show standard deviation.

Response times were significantly faster in the physical pointing condition ($\bar{x}$ = 383.1 ms, *σ* = 40.7 ms) than in the button press condition ($\bar{x}$ = 515.8 ms, *σ* = 52.9 ms) when response onsets in the physical pointing condition were defined as starting at 5% of the subsequent peak velocity (*p* < 0.001). The means for each condition and participant are shown in Figure 4. Significant differences in response onsets between the two response conditions were observed up to a threshold of 53% of the subsequent peak velocity (*p* < 0.05; $\bar{x}$ = 474.3 ms, *σ* = 41.5 ms).

Response accuracies were very high with an average of only 0.24% and 7.99% false alarms to color changes indicating a standard stimulus in the button press and physical pointing condition, respectively. Incorrect responses to distractors revealed comparable tendencies with 1.13% and 7.47% false alarms for button presses and physical pointing responses, respectively. In cases of color changes indicating a target stimulus only 0.20% misses were observed for the button press condition and no incorrect responses at all (0%) in the physical pointing condition. While for both standard and distractor stimuli more incorrect responses were observed in the physical pointing condition, target stimuli were associated with less incorrect responses when participants had to point at the moving object. However, only 3 out of 12 participants committed errors in the physical pointing condition while eight participants committed errors in the button press condition. Due to the absence of incorrect responses in the majority of participants no further statistical analyses was conducted. Table 1 displays mean and standard deviations of response errors in all conditions.

**Table 1:** **Means and standard deviations of response errors for all conditions**.

	Physical pointing	Button press
Standard	$\bar{X}$ = 7.99%, σ = 0.2155	$\bar{X}$ = 0.24%, σ = 0.0036
Distractor	$\bar{X}$ = 7.47%, σ = 0.2407	$\bar{X}$ = 1.13%, σ = 0.0175
Target	$\bar{X}$ = 0.00%, σ = 0.0000	$\bar{X}$ = 0.20%, σ = 0.0067

### EEG Data

**Figure 5:**
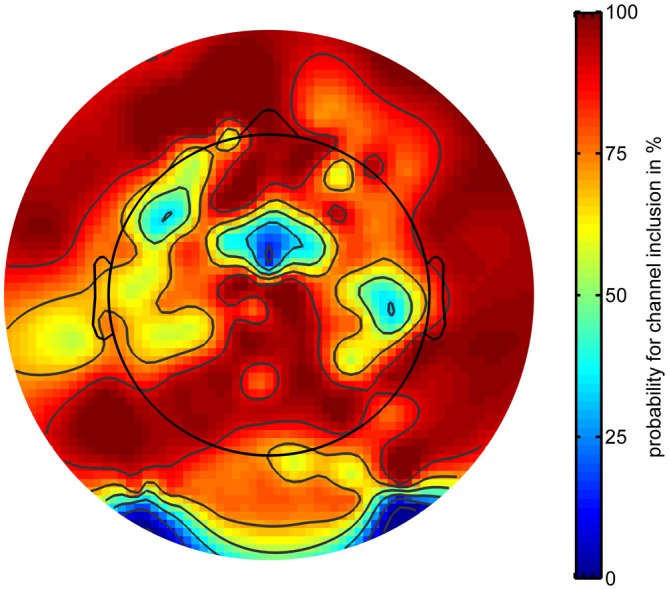
**Probability of electrodes to be included in subsequent analyses as a function of electrode location.** Warm (red) colors indicate higher probabilities.

Rapid volatile pointing movements were associated with increasing artifactual activity stemming from both physiological and mechanical sources. To correct for artifactual activity, the EEG signal was cleaned in the time and channel domain (see “Materials and Methods” Section). Cleaning in the channel domain revealed a specific topography for channels with a high probability to be removed. Figure 5 displays the probability for each channel to be included in the analysis plotted with respect to its scalp position.

Channels were most likely to be removed in five different regions of the montage with the highest likelihood of removal for channels located to the left and right posterio-inferior locations in the montage. One position over the midline located near Cz and two lateralized areas around FT7 and TP8 also showed a high likelihood of channel removal. On average, a subset of 24 channels were removed from the montage before further data analyses (range: 14–42).

### Event-Related Potentials on the Sensor Level

**Figure 6:**
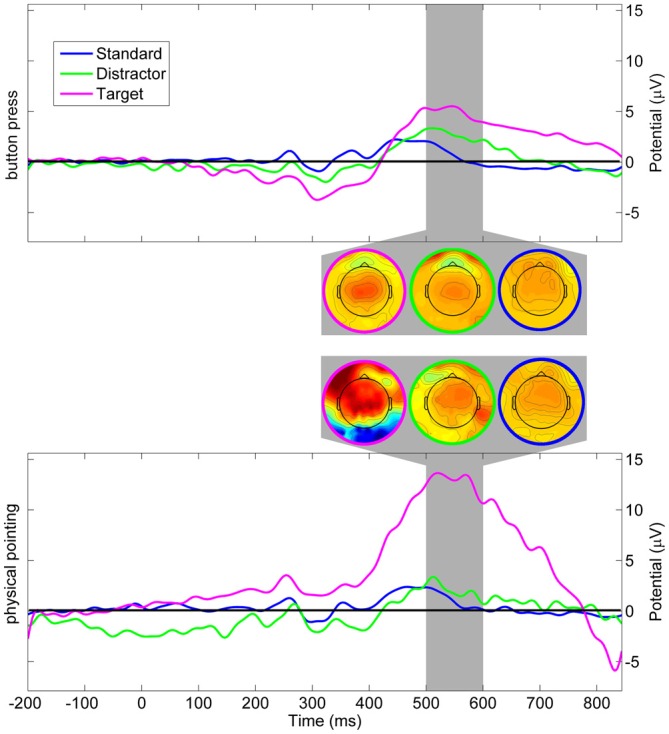
**Grand average event-related potential (ERP) at Pz’.** Upper panel displays scalp potentials in the button press condition, lower panel displays scalp potentials in the physical pointing condition. Blue: standard stimuli, green: distractor stimuli, magenta: target stimuli. The dark gray area displays the latency range of the mean topographic EEG maps for the different stimuli and response conditions.

Changes in the color of the moving sphere were associated with ERPs including a late positive complex at parietal sensors in the time range of the P3. Figure 6 displays ERPs with onset of color changes indicating standard, distractor, and target stimuli for the button press and the physical pointing condition for the electrode closest to the parieto-central electrode of the international 10–20-system (referred to as Pz’ in the following). To investigate differences in the P3 component measured at the scalp, mean amplitudes in the time range from 400 to 800 ms after a color change were submitted to a 2 (response condition) × 3 (stimulus type) repeated measures ANOVA. Greenhouse-Geisser corrected *p*-values are reported in case of non-sphericity. The results revealed a significant main effect of stimulus type (*F*_(2,22)_ = 8.58, *p* = 0.010, *η*^2^ = 0.343) and a tendency for the response condition (*F*_(1, 11)_ = 3.32, *p* = 0.084, *η*^2^ = 0.247) but no interaction effect (*F*_(2, 22)_ = 2.99, *p* = 0.208, *η*^2^ = 0.133). *Post hoc* HSD contrasts (Tukey, 1949) revealed that the P3 amplitude for targets in the pointing condition was significantly higher than for standards in both response conditions (all *p*s < 0.009) as well as distractors in the button press condition (*p* = 0.02). Comparing P3 amplitudes for targets and distractors in the pointing condition revealed only a trend towards significance (*p* = 0.09) and there was no significant difference between targets in the physical pointing and the button press condition (*p* = 0.14). There were no significant differences between any of the stimuli in the button press condition (all *p*s > 0.70).

While both response conditions were associated with increased P3 amplitudes for targets as compared to standard stimuli and distractors, the physical pointing condition demonstrated stronger amplitude increases in the time range of the P3 as compared to the button press condition. The stronger effect in the pointing condition could have been caused by increased processing demands or a generally higher alertness in a condition that required fast responses to a dynamically moving target. However, because the P3 component was located in a time window that also comprised participants pointing responses, increased P3 amplitudes might have been confounded with non-brain related processes. The rather strong jerks of the rapid pointing movements could have added mechanical artifacts induced by the movement. In addition, physical pointing at a moving target required constant coordination of eye, head, and arm movements that, due to volume conduction of the corneo-retinal potential and neck muscle activity, likely contributed to the P3 component at the sensor level. To further investigate to what extent signals from brain and non-brain sources like eye movements or muscle activity contributed to the sensor signal the correspondent independent component processes were analyzed.

### Contributions of Brain, Neck Muscle and Eye Movement Activity Related ICs

**Figure 7:**
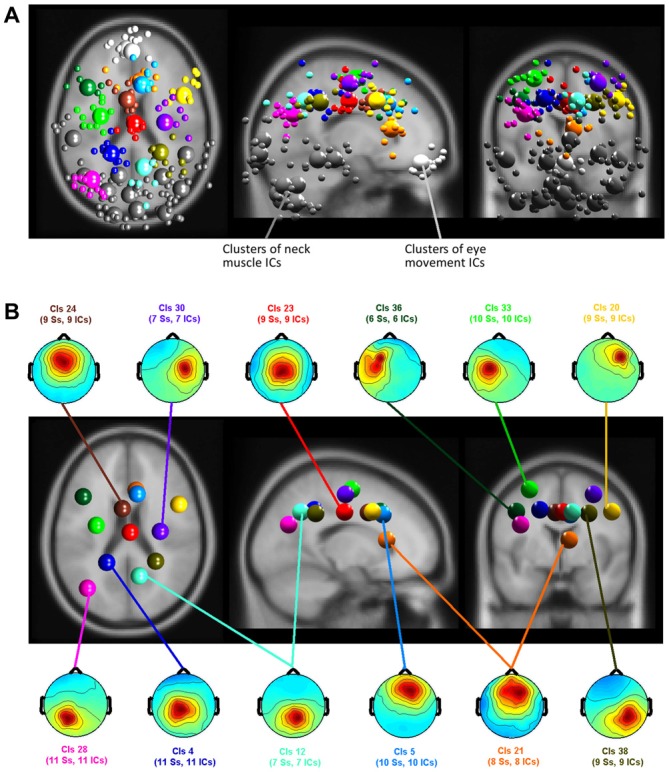
**(A)** Equivalent-dipole locations of neck muscle (dark gray), eye movement (bright gray) and brain-based independent components (ICs) and their cluster centroids (large spheres, corresponding color) projected to the horizontal, sagittal, and coronal views of the standard Montreal Neurological Institute (MNI) brain. **(B)** Mean projections to the scalp of brain-based IC cluster centroids with index (Cls #), number of participants (# Ss), and number of ICs (# ICs) for each cluster.

Clustering of ICs resulted in 26 clusters with cluster centroids located to the gray matter of the brain model or in regions of the model indicating eye movement or neck muscle activity. Figure 7 displays clusters of IC processes (smaller spheres) and their respective cluster centroids (larger spheres) reflecting brain dynamics, eye movement and muscle activity.

Back projection of event-related activity originating from different clusters to the sensors allowed for quantifying the contribution of brain and non-brain sources to the sensor P3 component. ERPs of clusters with a centroid located to the gray matter of the brain as well as clusters representing eye movement and neck muscle activity were selected for back projection. The absolute variance and the percent residual variance accounted for (pvaf) with respect to the P3 envelope was computed for all clusters for the time interval between 200 ms before stimulus onset to 1000 ms post stimulus. The pvaf of a specific cluster is defined as 1 − *R* where, *R* is the quotient of the absolute variance of the remaining clusters (after excluding the considered one) and the absolute variance of all clusters. The pvaf has an upper bound of 100% but can be negative if its projection to the scalp electrode cancels the projected signal of another cluster. This can happen in case ICs are spatially non-orthogonal. Pvaf values were used to estimate the relative share of certain clusters within one condition. Absolute variances, in contrast, allowed for comparing the contributions of one or more clusters to the sensor level in different response conditions where relative values could be misleading due to differences in overall absolute activity.

**Figure 8:**
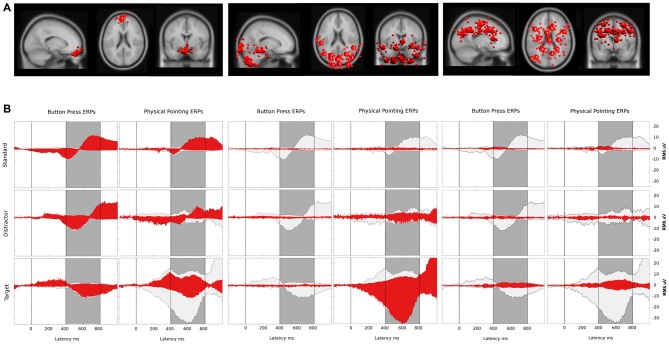
**(A)** Bigger spheres represent cluster centroids and smaller spheres display individual ICs representing horizontal and vertical eye movement activity (left), neck muscle activity (middle) and brain activity (right). The two clusters representing eye activity in the left column consisted of 12 ICs and 11 ICs, respectively. Neck muscle activity in the middle column is represented by 12 clusters comprising an average of 7.8 ICs (range: 4–10, *σ* = 1.5). Brain activity is represented in the right column by 12 clusters comprising on average 8.8 ICs (range: 6–11, *σ* = 1.5) from 12 participants. Cluster locations are projected onto the standard MNI brain volume and displayed in sagittal, horizontal, and coronal views. **(B)** Red: ERP contributions of clusters representing eye movement (left), neck muscle (middle), and brain (right) activity. Light gray: ERP envelopes of all 36 back-projected clusters. The dark gray area displays the latency range of the P3 component from 400–800 ms after a color change. The left and right columns display envelopes for the button press and the physical pointing condition, respectively, with rows displaying from top to bottom the different stimuli (standard, distractor and target).

Table 2 shows the resulting absolute variances and pvafs. Here, the total variance refers to all 36 clusters resulting from the clustering, while neck variance refers to 12 clusters indicating neck muscle activity, eye variance refers to two clusters contributing to horizontal and vertical eye movements, and brain variance to 12 clusters located to the gray matter of the brain. Figure 8 displays in gray the back-projected summed sensor signal envelope based on all brain, eye, and neck muscle clusters and in red from left to right the contribution of clusters accounting for eye movements, neck muscle activity, and brain activity, respectively.

**Table 2:** **Variance in μV^2^ in the −200 to 1000 ms time range for the total data and separately for clusters of eye movement, neck muscle, and brain activity**.

		Total variance	Eye variance (pvaf)	Neck variance (pvaf)	Brain variance (pvaf)
Standard	Physical pointing	2.39	1.58 (82.4%)	0.03 (3.6%)	0.17 (7.4%)
	Button press	3.45	2.12 (87.9%)	0.01 (-0.2%)	0.07 (3.8%)
Distractor	Physical pointing	4.46	1.26 (38.2%)	0.56 (12.0%)	0.53 (13.8%)
	Button press	3.04	2.71 (83.3%)	0.04 (-2.7%)	0.14 (8.2%)
Target	Physical pointing	19.32	1.98 (15.3%)	6.09 (56.6%)	1.33 (10.6%)
	Button press	3.17	1.62 (63.9%)	0.05 (3.6%)	0.43 (34.3%)

*Brackets show the corresponding pvaf in %. Columns are displaying values separately for the cluster combinations and rows display values to standard, distractor and target stimuli in the physical pointing and button press condition*.

### Relative Contribution of Clusters to the Envelope

#### Button Press Condition

The relative contributions to the ERP envelope for standard stimuli in the button press condition was high for eye movement activity, only marginal for neck muscle activity, and low for brain processes (eye: 87.9%, neck: −0.2%, brain: 3.8%). Decreasing contribution of eye movement activity and increasing contributions of brain processes was observed for distractor stimuli (eye: 83.3%, neck: −2.7%, brain: 8.2%) and target stimuli (eye: 63.9%, neck: 3.6%, brain: 34.3%).

#### Physical Pointing Condition

The contributions to the envelope of the ERP for standard stimuli in the pointing condition (eye: 82.4%, neck: 3.6%, brain: 7.4%) were similar to those in the button press condition with slightly stronger contributions of neck muscle activity and brain processes. This trend grew stronger for distractor stimuli (eye: 38.2%, neck: 12.0%, brain: 13.8%) with a pronounced drop in eye movement contribution. For targets neck muscle activity exceeded all other processes considerably (eye: 15.3%, neck: 56.6%, brain: 10.6%).

### Absolute Contribution of Clusters to the Envelope

#### Button Press Condition

In the button press condition the absolute variance of all non-brain and brain processes was relatively stable for standard (3.45 μV^2^), distractor (3.04 μV^2^) and target stimuli (3.17 μV^2^). The absolute contribution of clusters representing eye movements revealed 2.12 μV^2^ for standards, 2.71 μV^2^ for distractors, and 1.62 μV^2^ absolute variance for targets. For clusters with the equivalent dipole model of the cluster centroid located in or near regions of the head model indicative of neck muscles the absolute variance increased from standard (0.01 μV^2^) to distractor (0.04 μV^2^) and target stimuli (0.05 μV^2^). The same trend was observed for clusters representing brain activity contributing 0.07, 0.14, and 0.43 μV^2^ absolute variance for standard, distractor, and target stimuli, respectively.

#### Physical Pointing Condition

In the physical pointing condition the absolute variance of all non-brain and brain processes strongly increased from standard (2.39 μV^2^) and distractor (4.46 μV^2^) to target stimuli (19.32 μV^2^). The absolute contribution of clusters representing eye movements revealed lower values compared to the button press condition explaining 1.58 μV^2^ for standards, 1.26 μV^2^ for distractors, and 1.98 μV^2^, for targets. The absolute variance for clusters representing neck muscle activity increased from standard (0.03 μV^2^) to distractor (0.56 μV^2^) and target stimuli (6.09 μV^2^). A comparable pattern was observed for brain activity with the lowest absolute variance for standard (0.17 μV^2^) and distractor stimuli (0.53 μV^2^), followed by target stimuli (1.33 μV^2^).

In summary, the absolute variance and the increase in absolute variance for clusters representing brain and neck muscle activity were more pronounced in the physical pointing condition, with clusters representing neck muscle activity explaining by far the highest amount of the sensor envelope for target stimuli. In contrast, eye movement contributions were lower for standard and distractor stimuli in the physical pointing condition.

Compared to neck muscle and eye movement activity, the contribution of brain processes to the surface potential was relatively small in both response conditions demonstrating a prominent role of non-brain sources for sensor based ERP analyses during active movements of the head and upper torso.

To further investigate the brain dynamics accompanying target processing in the physical pointing as compared to the button press condition, all non-brain clusters were excluded and only brain-related activity was back projected to the sensor level.

### Relative Contributions of Brain Activity to the Sensor Event-Related Potential

Examining the grand average ERP from back-projecting all clusters representing brain activity revealed which clusters contributed most to the sensor level variance in the time window of the P3 component of the ERP. Table 3 displays the explained absolute and relative (pvaf) variance for the parietal and anterior cingulate cortex (ACC) clusters for each condition in the 400–800 ms time window. For pvafs and absolute variances of all brain clusters, see Supplementary Table 1.

**Table 3:** **Variance in μV^2^ in the 400–800 ms time range for all clusters contributing to brain activity and separately for the parietal and ACC clusters**.

		Total brain variance	Parietal variance (pvaf)	ACC variance (pvaf)
Standard	Physical pointing	0.32	0.004 (6.2%)	0.098 (68.6%)
	Button press	0.14	0.004 (3.6%)	0.055 (72.5%)
Distractor	Physical pointing	0.43	0.020 (11.7%)	0.094 (39.5%)
	Button press	0.25	0.010 (20.1%)	0.076 (58.8%)
Target	Physical pointing	3.11	0.954 (55.4%)	0.486 (0.3%)
	Button press	0.87	0.130 (38.2%)	0.084 (1.5%)

*Brackets show the corresponding pvaf in %. Columns are displaying values separately for the cluster combinations and rows display values to standard, distractor and target stimuli in the physical pointing and button press condition*.

The absolute variance of the sensor ERP explained by brain processes increased from standard (0.14 μV^2^) to distractor (0.25 μV^2^), and target stimuli (0.87 μV^2^) in the button press condition. The same trend was observed for the physical pointing condition with lowest absolute variance for standards (0.32 μV^2^) and distractor stimuli (0.43 μV^2^), followed by target stimuli (3.11 μV^2^). The amount of variance explained and the increase in explained variance was stronger in the physical pointing condition.

**Figure 9:**
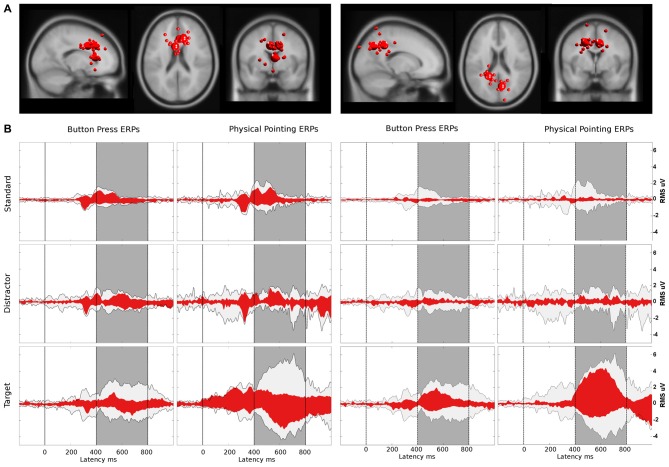
**(A)** Bigger spheres represent cluster centroids and smaller spheres individual ICs with the cluster centroid located in or near the anterior cingulate cortex (ACC; left) and the dorsal parietal cortex (right). Cluster locations are projected onto the standard MNI brain volume and displayed in sagittal, horizontal, and coronal views. One cluster, located in the left ventral ACC (talairach coordinates: *x* = −5, *y* = 9, *z* = 34, corresponding to BA 24/BA 32) consisted of nine ICs. A second cluster located near the right ventral ACC (*x* = 3, *y* = 24, *z* = 6, near to BA 24) comprised eight ICs and a third located near to the right dorsal ACC (*x* = 12, *y* = 26, *z* = 31, corresponding to BA 9/BA 32) comprised 10 ICs from 12 participants. For the clusters near the parietal cortex, one was located in the left parietal cortex (talairach coordinates: *x* = −19, *y* = −42, *z* = 39, corresponding to BA 31) and consisted of 11 ICs. A second cluster located in the posterior parietal cortex (*x* = 11, *y* = −66, *z* = 38, corresponding to BA 7) comprised seven ICs from 12 participants. **(B)** Red: ERP contributions of the clusters located in or near the ACC and the parietal cortex, respectively; light gray: ERP envelope computed by back-projecting all clusters located in the gray matter of the brain model. The dark gray area displays the latency range of the P3 component from 400–800 ms after a color change which was used for calculating corresponding pvafs. The left and right columns display envelopes for the button press and the physical pointing condition, respectively, with rows displaying from top to bottom the different stimuli (standard, distractor and target).

The general pattern observed for the contribution of all brain clusters was also observed for the backprojection of a subset of clusters with their centroids located in or near the anterior cingulate and parietal cortex. Three clusters (Cls 5, 21, and 24) representing brain activity in or near the ACC explained lower absolute variance for standard (0.098 μV^2^) and distractor stimuli (0.094 μV^2^) than for target stimuli (0.486 μV^2^) in the physical pointing condition. A different contribution was observed in the button press condition with increasing absolute variance for standards (0.055 μV^2^) to distractors (0.076 μV^2^), and targets (0.084 μV^2^). Because of the general increase in absolute variance in the target condition the relative contribution of the ACC clusters was considerably more pronounced for standard and distractor stimuli than for the target related P3 (see Figure 9). The relative contribution of the ACC clusters for standard stimuli was 68.6% and 72.5%, for distractor stimuli 39.5% and 58.8% and for target stimuli 0.3% and 1.5% in the physical pointing and the button press condition, respectively.

Parietal clusters explained increasing variance with the lowest contribution for standard stimuli (0.004 μV^2^), followed by distractor (0.010 μV^2^) and target stimuli (0.130 μV^2^) in the button press condition. This increase from standard to target was also observed for the physical pointing condition with 0.004, 0.020, and 0.954 μV^2^ for standard, distractor and target stimuli, respectively. With 55.4% in the physical pointing condition and 38.2% in the button press condition the two parietal clusters contributed the most to the P3 signal for target stimuli. The right panel of Figure 9 displays two clusters located in or near the parietal lobe and their summed backprojected ERP activity relative to the envelope of all ICs representing brain activity.

Beyond the contribution of the described clusters located in or near the ACC and parietal lobe, other clusters also contributed to the sensor envelope for target stimuli in the P3 time range in the physical pointing condition. These clusters were located in or near the junction of the left parietal and occipital cortex (*x* = −40, *y* = −73, *z* = 27 corresponding to BA 39/BA 19) explaining 38.3%, the right motor and premotor cortex (*x* = 40, *y* = −6, *z* = 54, corresponding to BA 6/BA 4) explaining 14.8%, and the left dorsolateral prefrontal cortex (*x* = −43, *y* = 22, *z* = 31, corresponding to BA 9) explaining 9.8% of variance of the sensor envelope (see Supplementary Table 1 for additional cluster contributions in the button press condition).

### The Contribution of Brain Activity to the P3 at Pz’

**Figure 10:**
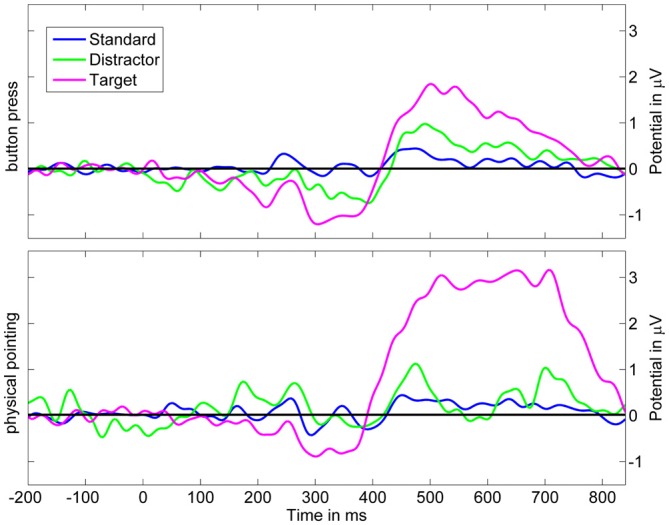
**Grand average ERP at Pz’ based on backprojection of clusters representing brain activity.** Upper row displays scalp potentials for the button press condition, the lower row for the physical pointing condition. Blue: standard stimuli, green: distractor stimuli, magenta: target stimuli.

To analyze the brain dynamic contribution to the maximum of the P3 at the central parietal electrode, only ICs with their equivalent dipole model located to the gray matter of the brain were back projected to Pz’ (see Figure 10). The resulting summed activity was analyzed with respect to the response condition and stimulus type. To this end mean ERP amplitudes at Pz’ were calculated for a time window ranging from 400 to 800 ms after a color change of the sphere and tested for statistical differences using a 2 × 3 repeated measures ANOVA with the factors response condition (physical pointing vs. button press) and stimulus type (standard, distractor, target). Greenhouse–Geisser correction was performed in cases where the assumption of sphericity was violated.

The analysis revealed a significant main effect of the response condition (*F*_(1,11)_ = 11.70; *p* = 0.006; *η*^2^ = 0.515) and stimulus type (*F*_(2,22)_ = 16.04; *p* = 0.001; *η*^2^ = 0.593). The interaction of both factors was also significant (*F*_(2,22)_ = 12.47; *p* = 0.003; *η*^2^ = 0.531). *Post hoc* HSD contrasts revealed that P3 amplitudes were significantly higher for targets in the pointing condition as compared to standards and distractors in the pointing condition (all *p*s < 0.001) as well as for standards, targets, and distractors in the button press condition (all *p*s < 0.001). In the button press condition the P3 amplitude was significantly higher for targets than for standard stimuli (*p* < 0.02) but did not differ from distractor stimuli (*p* > 0.19).

## Discussion

In the present study, a visual oddball paradigm was used to investigate the feasibility of MoBI during volatile rapid movements. The systematic manipulation of response requirements to color changes of a dynamically moving object allowed for a direct comparison of ERPs during simple button presses and active physical pointing. Whereas earlier studies demonstrated that treadmill walking introduces comparatively more eye movements than neck muscle activity (Gramann et al., 2010a) the impact of neck muscle activity was much stronger in the present study with non-stereotyped pointing movements accompanying a wide range of different velocities and movement directions. To react properly in the physical pointing condition participants were requested to move fast and accurately requiring continuous tracking of the stimulus accompanied by eye and head movements and, whenever a target appeared, rapid arm and head movements integrating visual information from the dynamically moving object to intercept the target. This mimicked the fundamental difference between traditional imaging approaches using simple button responses and the MoBI approach allowing for natural interaction with the environment.

The present study revealed important new insights into the brain dynamics accompanying physical interaction with a moving object. Firstly, the study clearly demonstrated that MoBI is feasible for recording and analyzing embodied cognitive processes and the accompanying brain/body dynamics during volatile rapid movements in a realistic 3-D environment. Secondly, applying blind source separation methods to the EEG signals recorded during the visual oddball paradigm allowed for separating and clustering ICs corresponding to neck muscle activity, eye movements or brain processes. This way it was possible to analyze the contribution of different clusters to the scalp signal revealing strong activity of neck muscles during the physical pointing response resulting from head orientation changes and compensation of shoulder and arm movements during pointing. Thirdly, movement onsets and corresponding reaction times in the physical pointing condition demonstrated significantly faster response onsets as compared to the button press condition. Fourthly, analysis of the data in the time range of the P3 component revealed a clear P3 in both response conditions at the sensor level as well as the level of cluster activity. This manifested in significantly higher mean ERP amplitudes for target stimuli as compared to standard stimuli as well as increasing absolute variance for standard, to distractor, and target stimuli in both response conditions. Finally, back-projecting all brain-related clusters to the centro-parietal sensor showed significantly higher P3 amplitudes for target stimuli in the physical pointing condition compared to the button press condition. This finding indicates different brain dynamics for different behavioral states and has far-reaching implications in the field of Neuroergonomics.

### Natural Cognition and the Contribution of Brain and Non-Brain Sources

During physical interaction with a dynamically moving object, non-brain sources stemming mainly from eye movements and neck muscle activity as well as mechanical artifacts strongly diminished the observable fraction of brain activity recorded on the scalp and avoided meaningful analysis of sensor-based potentials without further preprocessing. However removing all ICs not associated with brain activity allowed for analyzing the P3 component and the contribution of different clusters to its time course revealing the following findings.

Clusters contributing to the sensor P3 component were mostly in line with the results of previous studies. As in Makeig et al. (2004), central parietal, motor and occipital processes contributed to the P3 with the largest contribution of parietal clusters to the onset of target stimuli. The contribution of brain processes located near or in the ACC was in line with previous findings using an oddball paradigm during treadmill walking (Gramann et al., 2010a).

The explained variance of brain related sources increasing from standard, to distractor, and target stimuli were found in both response conditions, with a stronger effect in the physical pointing condition. This is consistent with the assumption that a potential physical interaction with the environment requires additional cognitive and motor processes and thus leads to higher computational effort. In the present study it was necessary to track the position and movement direction of the relevant object and body parts required for orienting to and interacting with the stimulus. Physical interaction with target stimuli required action planning, execution, and control. Those processes were not required for frequent standard and rare distractor stimuli reflected in smaller amplitudes and lower variance in clusters reflecting brain processes. However, distractors attracted more attention and potentially triggered an initial response. This response had to be suppressed resulting in additional inhibitory processes and accompanying brain activity as indicated by higher variance for distractor stimuli than for standard stimuli.

Clusters representing neck muscle activity also accounted for increasing variance from standard, to distractor and target stimuli in both conditions. The increase was stronger in the physical pointing condition where a correct response to the target required a pointing movement comprising movement of the head, shoulder and arm. Those movements were accompanied by strong neck muscle activity as observed for target stimuli in the physical pointing condition. Since the readiness to act was very high, as indicated by faster response times and the absence of any misses, it is likely that rare distractor stimuli caused the initiation of response movements. Even in case the response was subsequently inhibited for distractor stimuli, response initiation would be reflected in higher neck muscle contribution to distractor than to standard stimuli.

Finally, clusters representing eye movements explained more variance in the sensor signal in the button press as compared to the physical pointing condition for standard and distractor stimuli. One possible explanation is that in the physical pointing condition head alignment to the stimulus position facilitated physical movements in that direction. As a consequence of increasing head movements during stimulus tracking, less eye movements were required for keeping the moving stimulus in the visual field. However, since the sphere kept moving after the color change, successful pointing movements to targets required an ongoing prediction of its future position. This caused extended coordination of eye and arm movement resulting in an increase of variance explained by eye movements. In the button press condition a simple button press was sufficient to respond to a color change requiring no further coordination of eye movement and physical response. In addition, the visual stimulus stopped moving after response execution rendering stimulus tracking unnecessary. This would have resulted in a decrease of corresponding variance for target trials compared to distractor and standard trials in both conditions.

### Limits of MoBI

The present study required continuous head and eye movements during stimulus tracking causing electrical potentials on the surface electrodes superposing the EEG signal. This happened especially for target stimuli in the physical pointing condition where a correct response demanded arm movements accompanied by strong jerks associated with increased neck muscle activity. Subsequently no significant mean ERP difference was found on the scalp electrodes in the P3 time range between physical pointing and button press for target stimuli. Volume conducted non-brain activity in the recorded EEG signal is an inevitable consequence of active movements of the participants. Using ICA for separating brain related from non-brain related activity and back projecting the former to the Pz’ revealed the expected differences between those conditions. Thus, MoBI proved feasible for analyzing event-related EEG dynamics of participants performing rapid pointing movements in a realistic 3-D environment.

However some caveats were identified in this study indicating potential constraints of the MoBI approach for investigating natural movements. These included an increase of artifact contaminated trials and channels as well as higher residual variances compared to EEG studies with stationary participants that are not allowed to move their heads.

A relatively high number of trials had to be removed due to inconsistencies between markers written online during the experiment and those derived afterwards from the velocity profiles. Thus the amount of considered data was decreased impeding statistical analysis especially in the physical pointing condition. Future MoBI experiments will need a setup fully covered with fixed cameras minimizing the risk of LED occlusions and camera movement causing such inconsistencies.

Related to the reduced number of trials due to technical problems, the impact of movement-related mechanical artifacts like cable sway was reflected in a specific distribution of the probabilities for channels to be removed. Jerks and micromovements of the electrodes over the skin surface associated with fast response movements led to impedance changes with strong artifactual activity affecting the outmost neck electrodes in the posterio-inferior regions. The central midline area as well as the two lateral regions over the scalp in contrast were most likely affected by cable pull during head movements due to the cable routing over the scalp to the back. This is one likely explanation for the high rejection rate of those electrodes. The lateral regions near the mastoid processes were predestined for bad contact with high impedance leading to artifactual activity due to the fit of the cap. For further MoBI experiments a redesign of electrode attachment or cable-free electrodes has to be considered to increase the number of channels that can be analyzed.

Finally, in contrast to non-MoBI studies the ICA results revealed many ICs with large residual variances that would not be included into the clustering process applying standard selection criteria (e.g., Gramann et al., 2010b). However muscle activity and eye movements originate in regions of the head that are usually not included in the head model for source reconstructions rendering it difficult to calculate suitable dipole models. Moreover, muscle contraction causes tissue movements which could result in dipole displacement. Thus, although in general higher residual variance is associated with decreased result accuracy, dipoles with relatively large residual variances were included in the analysis. A future improvement would be the introduction of forward models including neck muscles and their contraction profiles as additional parameter for the inverse solution.

### Implications on Neuroergonomics Research

The faster response onsets in the physical pointing condition might be the consequence of another brain dynamic state caused by the need of physical interaction with the stimulus. In addition, the physical pointing condition might have led to increased motivation and more fun for this response format as reported by the participants after the experiment. However, using movement and velocity profiles for the purpose of additional brain activity analysis requires the definition of corresponding features that are widely used and accepted. Defining the movement onset as a fixed percentage of the corresponding maximum velocity as in this study was only one possible solution. Other criteria might be useful in different contexts like a fixed absolute velocity or acceleration value which would be independent of individual movement differences. A general definition should be discussed and established to increase comparability of experimental results in the field of Neuroergonomics and for MoBI research in general. Importantly, comparing different onset criteria starting from 5% of the corresponding peak velocity up to 53% of the corresponding peak velocity still indicated faster responses in the physical pointing condition as compared to button presses. Whether this was simply due to the fact that participants enjoyed the physical response format or whether interception of a dynamically moving objects was associated with a generally different behavioral and brain dynamic state will have to be investigated in future experiments.

There are clear arguments in favor of state differences in brain dynamics depending on the behavioral state. Introducing a task that requires large volatile movements not only produces muscle activity and eye movements but also requires additional processes that allow for movement planning, control and execution. This additional processes will be reflected in changes in brain dynamics. Moreover, the oddball paradigm required constant attention directed towards a sphere that moved within the borders of a large screen in front of participants. In addition, the physical pointing condition necessitated the prediction of the targets’ movement to integrate this information with proprioceptive information about position and orientation of the arm and hand for concurrent dynamic motor planning and execution. Continuous observation and integration of environmental aspects with complex motor programs causes higher computational effort and can be assumed to lead to different brain dynamic states compared to passive observation. This is indicated by the significantly increased amplitude of the P3 and faster response onsets in the physical pointing condition compared to the button press condition. It would be important for future investigations to analyze the impact of stimulus speed on the brain dynamic state since higher speeds increase task difficulty and thus affect head and eye movement velocities.

This has significant implications for Neuroergonomics investigating the brain at work, especially in case the working environment requires physical interaction with a dynamic system. The physical pointing task required the participants to actively interact with their environment using fast, precise movements of the upper torso and the arm and hand. This generalizes to a wide range of working tasks where people have to manipulate objects as, for example, in assembly-line work or construction trade. Future studies might investigate the brain dynamics underlying spatially extended movements with different velocities including team sports or spatial orientation with or without navigation assistance. Studying the brain activity in the described work settings could provide valuable insights into the cognitive processes and the limits of the cognitive system and thus allow for suggestions how to increase system safety. For example, the degree of interaction seems to be one factor improving working environments by influencing motivation, reaction time and task complexity. Another factor to be considered is the body posture since active movement is naturally associated with an upright posture whereas cognitive neuroscientists still investigate sitting or lying participants. Changes in brain dynamics due to different body postures could have an impact on result quality and information processing speed.

Investigating the human brain dynamics accompanying physical interaction with dynamically moving objects for the first time, this study clearly demonstrated that it is possible to record and analyze EEG activity during volatile rapid movements. Thus, future MoBI studies examining the mentioned aspects will have an important impact on Neuroergonomics specifically and cognitive neuroscience in general.

## Author Contributions

KG and EJ have been actively involved in the experimental design. EJ recruited experimental participants, acquired and analyzed the data. KG participated in the data analysis. All authors contributed to the interpretation of the data, revised the manuscript and approved the final version to be published. EJ and KG agree to be accountable for all aspects of the work and ensure that questions related to the accuracy or integrity of any part of the work are appropriately investigated and resolved.

## Conflict of Interest Statement

The authors declare that the research was conducted in the absence of any commercial or financial relationships that could be construed as a potential conflict of interest.

## Footnotes

^1^http://www.sccn.ucsd.edu/eeglab
