# Supplementary figures and images for: Mobile Brain/Body Imaging (MoBI) of Physical Interaction with Dynamically Moving Objects
Source: Front Hum Neurosci. 2016 Jun 27;10:306. doi: 10.3389/fnhum.2016.00306 (PMC4921999; doi:10.3389/fnhum.2016.00306)

eye: [3, 6] + [27, 32]

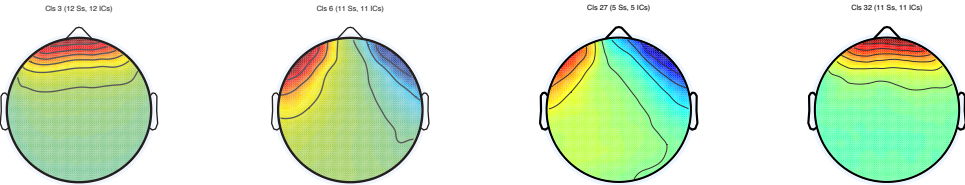

neck: [7, 9, 10, 11, 14, 19, 22, 26, 34, 37]

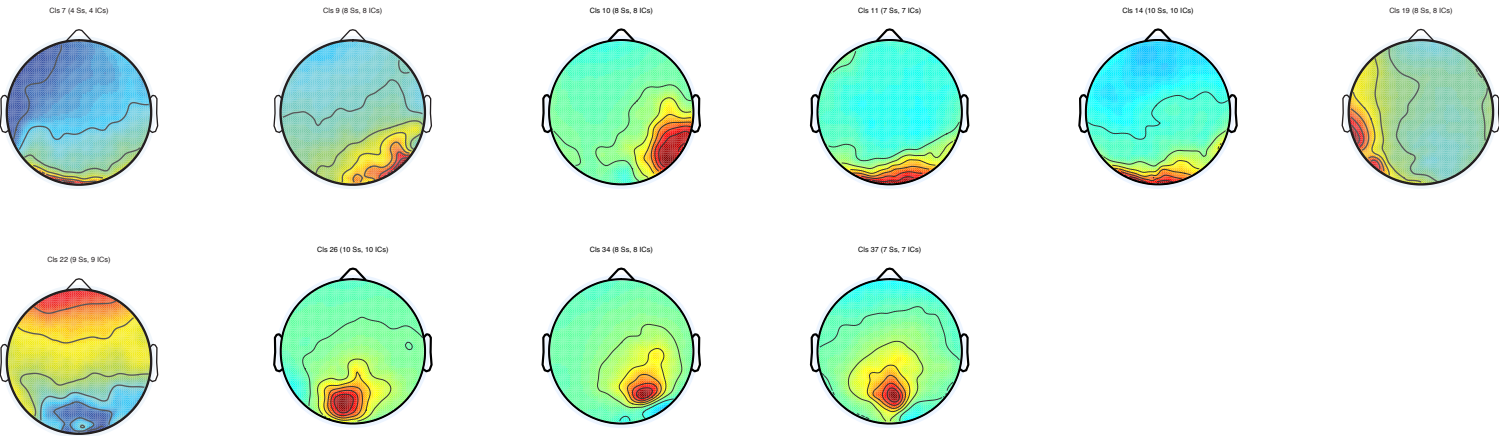

brain: [4, 5, 12, 20, 21, 23, 24, 28, 30, 33, 36, 38]

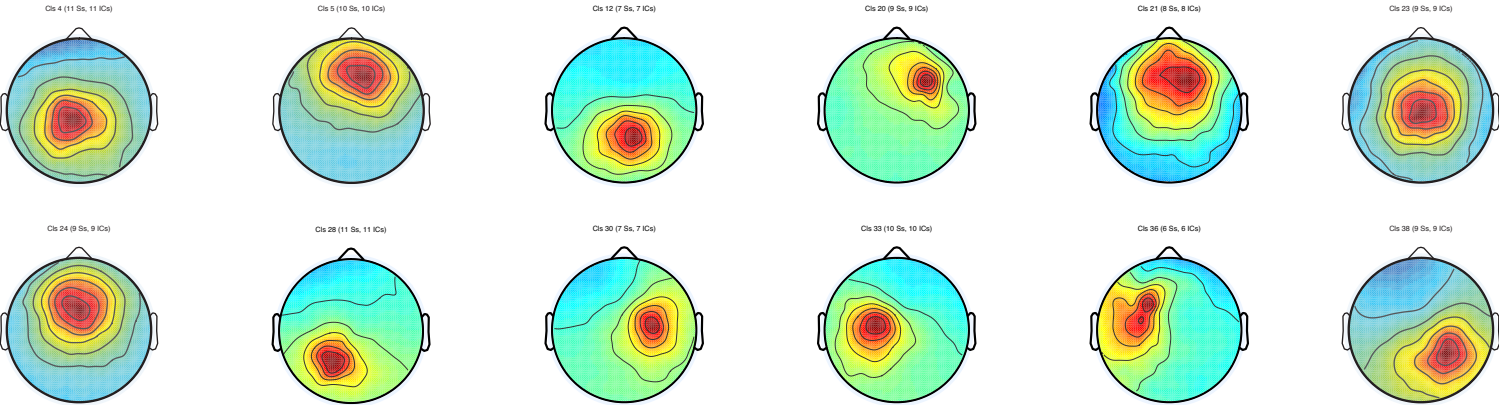

residual clusters: [8, 13, 15, 16, 17, 18, 25, 29, 31]

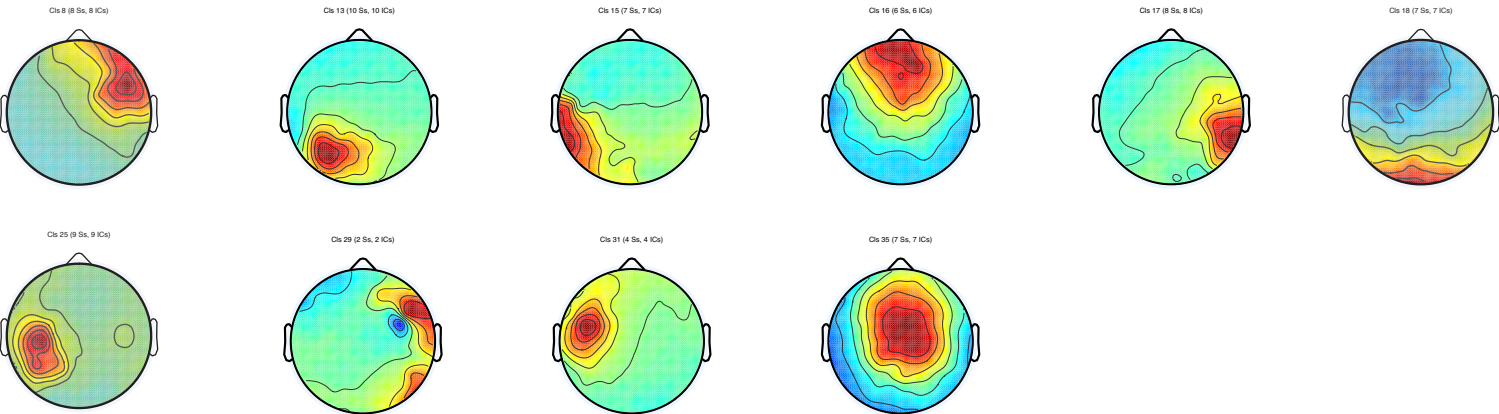

Supplement: Supplementary file 1 [file Image_1.pdf]

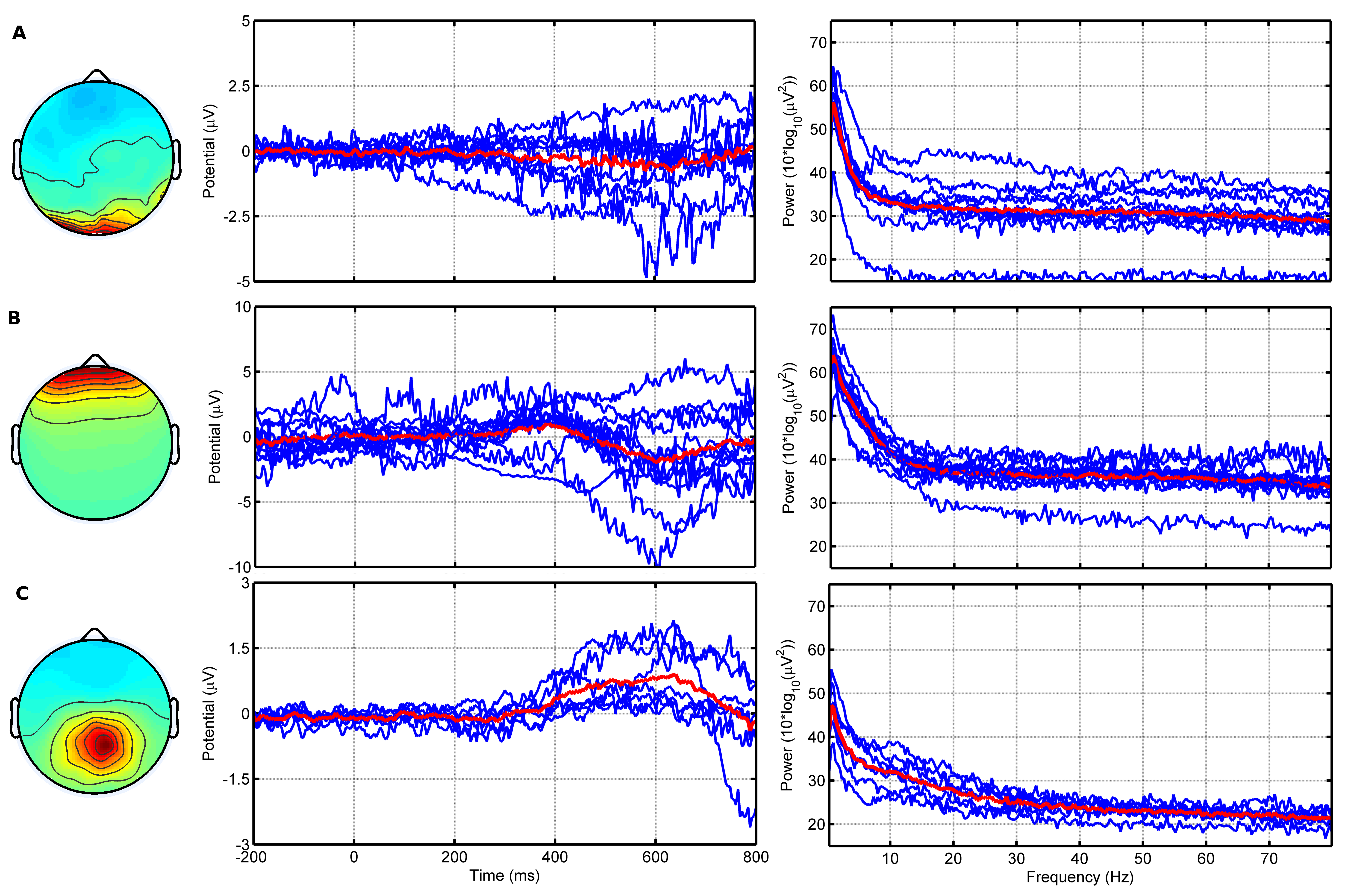

Supplement: Supplementary file 2 [file Image_2.tif]
