# Supplementary material for: Mobile Brain/Body Imaging (MoBI) of Physical Interaction with Dynamically Moving Objects
Source: Front Hum Neurosci. 2016 Jun 27;10:306. doi: 10.3389/fnhum.2016.00306 (PMC4921999; doi:10.3389/fnhum.2016.00306)
Supplement: Supplementary file 3 [file Table_1.docx]

Supplementary Material

Mobile Brain/Body Imaging (MoBI) of physical interaction with dynamically moving objects

**Evelyn Jungnickel^1^*, Klaus Gramann^1,2^**

^1^ Biological Psychology and Neuroergonomics, Institute of Psychology and Ergonomics, Department of Psychology and Ergonomics, Berlin Institute of Technology, Berlin, Germany

^2^ Center for Advanced Neurological Engineering, University of CA, San Diego, USA

*** Correspondence:** Evelyn Jungnickel: Evelyn.Jungnickel@tu-berlin.de

# Supplementary Data

## Supplementary Tables

**Supplementary Table 1.** Variance in µV^2^ in the 400 - 800 ms time range for all single clusters contributing to brain activity. Brackets show the corresponding pvaf in%. Columns are displaying values separately for the cluster combinations and rows display values to standard, distractor and target stimuli in the physical pointing and button press condition.

|  | **Cluster:** | **4** | **5** | **12** | **20** | **21** | **23** |
| --- | --- | --- | --- | --- | --- | --- | --- |
| **Standard** | Physical pointing  Button press | 0.0041  (2.64%)  0.0024  (1.80%) | 0.0096  (14.9%)  0.0084  (17.0%) | 0.0020  (2.8%)  0.0014  (2.2%) | 0.0007  (5.0%)  0.0006  (5.4%) | 0.0151  (30.1%)  0.0069  (23.8%) | 0.0140  (26.0%)  0.0045  (0.8%) |
| **Distractor** | Physical pointing  Button press | 0.0054  (1.0%)  0.0041  (7.6%) | 0.0386  (24.4%)  0.0258  (31.8%) | 0.0121  (11.4%)  0.0046  (13.2%) | 0.0013  (-1.2%)  0.0016  (-0.9%) | 0.0104  (-0.1%)  0.0050  (1.4%) | 0.0404  (6.3%)  0.0065  (5.1%) |
| **Target** | Physical pointing  Button press | 0.2187  (34.6%)  0.0625 (29.8%) | 0.0739  (14.4%)  0.0415  (7.5%) | 0.3171  (34.3%)  0.0251  (13.3%) | 0.0120  (-2.0%)  0.0018  (0.4%) | 0.1110  (-11.5%)  0.0185  (4.1%) | 0.0693  (3.9%)  0.0571  (27.8%) |
|  | **Cluster:** | **24** | **28** | **30** | **33** | **36** | **38** |
| **Standard** | Physical pointing  Button press | \| 0.0227  (39.3%) \| \| --- \| \| 0.0177  (48.2%) \| | \| 0.0040  (10.4%) \| \| --- \| \| 0.0041  (16.1%) \| | \| 0.0102  (11.0%) \| \| --- \| \| 0.0034  (9.3%) \| | \| 0.0012  (4.2%) \| \| --- \| \| 0.0020  (5.2%) \| | \| 0.0162  (12.3%) \| \| --- \| \| 0.0019  (4.5%) \| | \| 0.0061  (8.3%) \| \| --- \| \| 0.0050  (22.5%) \| |
| **Distractor** | Physical pointing  Button press | \| 0.0281  (19.1%) \| \| --- \| \| 0.0336  (30.1%) \| | \| 0.0089  (4.7%) \| \| --- \| \| 0.0099  (6.5%) \| | \| 0.0081  (10.8%) \| \| --- \| \| 0.0069  (14.8%) \| | \| 0.0051  (3.6%) \| \| --- \| \| 0.0033  (6.7%) \| | \| 0.1230  (37.8%) \| \| --- \| \| 0.0067  (2.8%) \| | \| 0.0448  (5.9%) \| \| --- \| \| 0.0203  (30.5%) \| |
| **Target** | Physical pointing  Button press | \| 0.6261  (-13.1%) \| \| --- \| \| 0.0523  (-13.3%) \| | \| 0.3347  (38.3%) \| \| --- \| \| 0.0186  (7.5%) \| | \| 0.1179  (14.8%) \| \| --- \| \| 0.0319  (20.9%) \| | \| 0.0205  (5.8%) \| \| --- \| \| 0.0167  (4.1%) \| | \| 0.0385  (9.8%) \| \| --- \| \| 0.0391  (23.8%) \| | \| 0.0776  (5.8%) \| \| --- \| \| 0.0552  (25.8%) \| |

## Supplementary Figures

**Supplementary Figure 1**

Scalp maps of clustered ICs from all participants with different origins, e.g. eye, neck muscles, and brain.

**Supplementary Figure 2**

**(A)** muscle, **(B)** eye, and **(C)** brain cluster including (from left to right) scalp maps, ERPs, and spectra of the ICs comprised for targets in the pointing condition.
